# Genetic and Environmental Effects on the Abdominal Aortic Diameter Development

**DOI:** 10.5935/abc.20150140

**Published:** 2016-01

**Authors:** Adam Domonkos Tarnoki, David Laszlo Tarnoki, Levente Littvay, Zsolt Garami, Kinga Karlinger, Viktor Berczi

**Affiliations:** 1 Department of Radiology and Oncotherapy - Semmelweis University, Budapest - Hungary; 2 Central European University, Budapest - Hungary; 3 Houston Methodist DeBakey Heart & Vascular Center - The Houston Methodist Hospital, Houston, TX - USA

**Keywords:** Aorta, Abdominal / genetics, Heredity, Atherosclerosis, Risk Factors

## Abstract

**Background:**

Configuration of the abdominal aorta is related to healthy aging and a variety of disorders.

**Objectives:**

We aimed to assess heritable and environmental effects on the abdominal aortic diameter.

**Methods:**

114 adult (69 monozygotic, 45 same-sex dizygotic) twin pairs (mean age 43.6 ± 16.3 years) underwent abdominal ultrasound with Esaote MyLab 70X ultrasound machine to visualize the abdominal aorta below the level of the origin of the renal arteries and 1-3 cm above the bifurcation.

**Results:**

Age- and sex-adjusted heritability of the abdominal aortic diameter below the level of the origin of the renal arteries was 40% [95% confidence interval (CI), 14 to 67%] and 55% above the aortic bifurcation (95% CI, 45 to 70%). None of the aortic diameters showed common environmental effects, but unshared environmental effects were responsible for 60% and 45% of the traits, respectively.

**Conclusions:**

Our analysis documents the moderate heritability and its segment-specific difference of the abdominal aortic diameter. The moderate part of variance was explained by unshared environmental components, emphasizing the importance of lifestyle factors in primary prevention. Further studies in this field may guide future gene-mapping efforts and investigate specific lifestyle factors to prevent abdominal aortic dilatation and its complications.

## Introduction

The size of the aorta decreases with distance from the aortic valve in a tapering fashion, the normal diameter of the descending aorta being defined as < 1.6 cm/m^2^, and that of the abdominal aorta, less than 3.0 cm.^1,2^ Configuration of the abdominal aorta can be related to healthy ageing, exercise, and/or a variety of disorders, such as hypertension, aneurysm, dissection or rupture.^1,2^ The increase of the aortic size is continous during life, the normal expansion rate is about 1-;2 mm/year and involves all segments.^3^ The ageing of the aorta is accompanied by a loss of compliance and an increase of wall stiffness caused by structural changes, including an increase in the collagen content, formation of intimal atherosclerosis with calcium deposits, smooth muscle activation, matrix degradation, cystic media necrosis, upregulation of proteolytic pathways and oxidative stress.^1,2,4^ It is also known that certain genetic diseases, such as abdominal aortic aneurysm formation, are associated with aortic geometry.^4,5^ The relationship between the increasing aortic size and the risk of spontaneous rupture or dissection has been well documented.^6^ Although familiar accumulation of these abdominal aortic aneurysms has been reported by several studies, the extent of genetic determination over the abdominal aortic geometry is still scarce. Although the prevalence of the diseases related to the abdominal aortic diameter (e.g., atherosclerosis, aortic aneurysm) has increased in the past decades due to lifestyle changes and various risk factors, no study has investigated whether the variation of the abdominal aortic diameter is due to genetic or environmental differences. If heritable effects are important, studies are necessary to identify specific genetic markers that determine abdominal aortic size. On contrary, if environment plays a greater role, an emphasis should be put on lifestyle interventions in order to influence the development of the abdominal-aortic-diameter-related diseases. Accordingly, the objective of this study was to assess the extent of genetic and environmental effects on the abdominal aortic diameter.

## Methods

### Participants and study design

From the Hungarian Twin Registry, 114 healthy Caucasian twin pairs [69 monozygotic (MZ), 45 same-sex dizygotic (DZ) twin pairs] above 18 years of age (mean age 43.6 ± 16.3 years) were selected and recruited for an abdominal ultrasound in the Department of Radiology and Oncotherapy of the Semmelweis University in 2009 and 2010^7^. We excluded opposite-sex DZ twin pairs to avoid bias of the heritability estimates in the presence of gender-specific or X-chromosome effects. Pregnant subjects were excluded from the study. Patients with atherosclerotic disease or other causes of aortic stenosis were not excluded from the analysis. Instead of genotyping for zygosity classification, we used a multiple­choice self­reported seven-part questionnaire.^8^ Risk factors, history of cardiovascular diseases and smoking habits were recorded on a questionnaire. All participants gave informed consent. This study was approved by the Ethical Committee of the Semmelweis University and was conducted in full compliance with regulations of the Declaration of Helsinki.

### Limited abdominal ultrasonography

A limited abdominal ultrasonography was performed using B-mode and Doppler ultrasound in order to visualize the abdominal aorta below the level of the origin of the renal arteries and 1-3 cm above the bifurcation, equipped with a curved array transducer (1-;8 MHz, CA431, Esaote MyLab 70X Vision, Esaote, Genova, Italy). Standardized digital images of the aorta were recorded in supine position, and transversal plane images were applied for axial views. The gray-scale amplification gain, the time-gain compensation curve, and focus number were adjusted to acquire the best images of the aorta. The examinations were performed by the same radiologist. Trackball was used in order to set the best image of the aorta in systole, and the largest aortic diameter was measured in the transverse plane by electronic calipers at the time of scanning.

### Statistical analysis

The SPSS Statistics 17 (SPSS Inc., Chicago, IL, USA) was used for descriptive analysis and comparison of MZ and DZ subsamples by independent samples t-test. Pearson correlations were calculated between aortic diameters and continuous variables. Aortic diameter parameters showed a normal distribution.

A descriptive estimate of the genetic influence in MZ and DZ pairs was calculated using the within-pair co-twin correlations corrected for the twins' age and gender. In twin analysis, substantially higher MZ co-twin correlation (compared to DZ correlations) suggests heritability, while similar co-twin correlations imply that shared environmental components drive the variance more strongly. Higher DZ similarities compared to MZ twins suggest that unshared (unique) environmental factors are responsible for the trait. Based on similarities between MZ and DZ twins, structural equation modeling (A-C-E model) was carried out with Mplus Version 6.1 (Muthén & Muthén, Los Angeles, CA, USA)^9^ in order to decompose the variance into additive genetic effects (A), and common (or shared) environmental (C) and unique (or unshared) environmental (E) effects.^10^ Empirical confidence intervals were calculated with a Bollen-Stine Bootstrap.^11^ All inferential statistics was estimated using full information maximum likelihood. Nested models were compared using likelihood-ratio and χ^2^ tests, and Akaike Information Criteria model selection was performed according to the principle of parsimony. P value < 0.05 was considered significant.

## Results

### Descriptive analysis

There were no significant differences between MZ and DZ twins in risk factors, history of diseases and clinical characteristics. Thirty-nine percent of the study sample was comprised of males. The MZ and DZ groups were formed by 32 males and 106 females, and 30 males and 60 females, respectively. Hypertension, hypercholesterolemia and diabetes were present in 32%, 22% and 6%. Active individuals represented 17% of the sample, and ex-smokers, 14%. The mean aortic diameters below the origin of the renal arteries and above the level of the aortic bifurcation were 1.5 ± 0.2 cm and 1.4 ±0.2 cm, respectively. There was no significant difference between MZ and DZ twins regarding the aortic diameter below the level of the origin of the renal arteries, but DZ twins had significantly smaller aortic diameter above the bifurcation (1.4 ± 0.2 vs. 1.3 ± 0.2 cm, p < 0.005). There was no abdominal aortic aneurysm among the subjects. There was no significant correlation between the aortic diameters and body mass index, weekly alcohol intake, cigarette smoking, diabetes and hypercholesterolemia. Hypertensive subjects had significantly larger aortic diameter below the level of the renal artery origin (1.6 ± 0.2 vs. 1.5 ± 0.2, p < 0.05), but there was no similar relationship above the level of the aortic bifurcation.

### Univariate analysis

Monozygotic co-twin correlations were higher than DZ ones, indicating that age- and sex-adjusted heritability of the abdominal aortic diameter below the level of the origin of the renal arteries was 40% [95% confidence interval (CI), 14 to 67%], and that above the aortic bifurcation was 55% (95% CI, 45 to 70%) (Table 1). None of the aortic diameters showed common environmental effects, but unshared environmental effects were responsible for 60% and 45% of the traits, respectively.

**Table 1:** Parameter estimates for additive hereditary (A), common environmental (C) and unique environmental (E) influences on aortic diameter by structural equation modeling

Dependent variable		AIC	BIC	-2LL	Χ^2^ difference	df difference	p value	rMZ	rDZ	A	95% CI	C	95% CI	E	95% CI
								0.413 (0.201, 0.630)	0.151 (-0.199, 0.436)						
Aortic diameter below the level of renal arteries*	A-C-E ‡	-57.544	-41.127	-69.54						0.40	0.14 - 0.67	0.00	0.00 - 0.38	0.60	0.36 - 0.80
	A-E	-59.544	-45.863	-69.54	0	1	1			0.40	0.20 - 0.62	0.00	0.00 - 0.00	0.60	0.38 - 0.80
	C-E	-57.339	-43.658	-67.34	5.628	1	0.018			0.00	0.00 - 0.00	0.30	0.14 - 0.48	0.70	0.52 - 0.86
								0.566 (0.366, 0.709)	0.209 (-0.069, 0.570)						
**Aortic diameter above the bifurcation†**	A-C-E	-79.736	-63.371	-91.736						0.55	0.29 - 0.72	0.02	0.00 - 0.56	0.45	0.29 - 0.64
	A-E ‡	-81.736	-68.099	-91.736	0	1	1			0.55	0.35 - 0.70	0.00	0.00 - 0.00	0.45	0.30 - 0.65
	C-E	-76.108	-62.472	-86.108	2.204	1	0.138			0.00	0.00 - 0.00	0.39	0.20 - 0.59	0.61	0.41 - 0.80

* 69 monozygotic, 45 dizygotic twin pairs;

† 68 monozygotic, 45 dizygotic twin pairs.

AIC: Akaike information criteria; BIC: Bayesian information criteria; LL: Log likelihood; Χ^2^: Chi-square test, based on model log likelihood comparative model fit test; rMZ: Saturated correlation between monozygotic twins; rDZ: Saturated correlation between dizygotic twin.

‡ best fitting model

Co-twin correlations of MZ (rMZ) and DZ (rDZ) twins offer basic insight on the heritability levels. Higher MZ correlations (vs DZ correlations) indicate a genetic effect, while similar rMZ and rDZ indicate shared environmental influence. 95% confidence intervals (CIs) are presented in brackets for all estimates. Univariate analyses are presented in which all components (A - additive genetic effects, C - common environmental effects, E - unique environmental effects) are estimated. Multiplying the numbers by 100 gives the variance in percentage. CIs not including 0 indicate significant estimates. Additionally reduced models are also shown (A-E or C-E), where the absent component is fixed at 0. Then we assess if these reduced models fit significantly worse (see p-value) than the full ACE model using a nested chi-square test. AIC and BIC offer additional evidence on which model is the most acceptable.

## Discussion

To our knowledge, our results demonstrate first the genetic effects on the abdominal aortic diameters in a healthy sample. This heritability was moderate, and a similarly moderate extent of variance was explained by unshared environmental components.

Only Cecelja et al^12^ have investigated the aortic dimensions in twins so far, and have reported that the highly heritable augmentation pressure in women is associated with the ratio of distal to proximal arterial diameters, but no heritability assessment of aortic diameters has been performed. The Strong Heart Study^13^ has investigated the heritability of echocardiographically derived aortic root diameters in the American Indian participants, and has reported an additive genetic contribution of 51%, which is comparable to our findings. However, the diameter of the abdominal aorta has never been assessed. Our findings underscore that there is a moderate role of genetic effects in the aortic diameter development, which may predispose to aortic dilatation. These findings indicate that, in half, genetic effects drive the ageing of the abdominal aorta, but in the other half of the variance it can be influenced by the lifestyle. This is a very important point because the loss of compliance, atherosclerosis and increase of aortic stiffness, which are related to aortic geometry and in which age-related increases in collagen synthesis potentially lead to dilatation, could be prevented with a healthy lifestyle.^14^ In addition, it has never been demonstrated that there is a segment-specific difference in the heritability: the aortic diameter above the level of bifurcation is more heritable than that below the level of the origin of renal arteries. We might speculate whether this finding could explain why atherosclerosis is more common before the bifurcation. It is known that hemodynamic disturbances, such as turbulent flow or low shear stress, associated with branching or high curvature, contribute to some of the localization of atherosclerosis.^15-18^ Segment-specific differences in heritability have been shown in case of carotid arteries indicating that inherited effects made a heterogeneous contribution to intima-media thickness by segment.^18^

In addition to the genetic mechanism, we reported that unique environmental factors, such as lifestyle, play a moderate role in determining aortic diameters. These risk and lifestyle factors have been extensively studied (smoking, hypertension, diabetes, obesity, etc.). According to some researchers, the programming of this mechanism begins already in foetal life and depends on mother's exposure to risk factors as well as via epigenetic modulation.^19^ Identification of risk factors, which have a 45-60% influence, rather proximally under the level of origin of renal arteries, would make the diagnosis more effective allowing possibility of detection of diseases in early stages and their prevention with lifestyle habit changing. The disease is believed to be connected with the lifestyle associated with high levels of oxidative stress and highly processed food.^20^

A long-term goal of the present study is to detect and map new polymorphic genes that influence variation in abdominal aortic size and thereby contribute to the development of asymptomatic subclinical aortic dilatation and dissection or rupture. In recent years, several (relatively few) genes related to abdominal aortic aneurysm formation have been reported. Susceptibility genes, rather than causal gene mutations, were suspected in aneurysms, particularly abdominal aortic aneurysms, which are genetically complex.^21^ A genome­wide study with infrarenal aorta diameter ≥ 30 mm or ruptured abdominal aortic aneurysm demonstrated that LDLR rs6511720 is associated with abdominal aortic aneurysm.^22^ A recent report showed that rs10757278 and rs1333049 on chromosome 9p21.3 are significantly associated with increased risk of abdominal aortic aneurysm in the Chinese population.^23^ However, genetic studies in healthy individuals are necessary and other population-based studies on genetic factors influencing abdominal aortic size need to be performed to identify specific genetic markers that determine abdominal aortic size. This information may lead to improvement in diagnostic and therapeutic strategies in the prevention of abdominal aortic dilatation and its complications.

Limitations of our study must be noted. No ECG gating was applied in our study, but systolic dilatation of the aorta was well identifiable in all cases. Second, aortic size measurements performed provide only a single static evaluation at one point in time. Older patients might demonstrate a more significant influence of environmental factors on aortic size, and alter the analysis of this study. Also, the relationship of genetic and environmental influence on aortic growth over time would be significantly more important than the influence over size at one point in time. In addition, ultrasonography has a weakness of inter-observer variability and limited visibility of aorta due to bowel gases and obesity, but in this study all participants were evaluated by the same radiologist and the aorta was well visible in all cases. Ultrasound was found to be relatively equal in the imaging of abdominal aorta compared to computed tomography.^24^ Additional limitation includes the relatively small number of participating DZ twins compared to usual twin studies, which may lead to statistical errors in the A-C-E model analysis by increasing the E variance.

## Conclusions

In summary, moderate heritability and its segment-specific difference of abdominal aortic diameter were shown in a healthy sample, which will guide future gene-mapping efforts. Unshared environmental factors were responsible for the other, moderate portion of the variance.
